# Anaphylaxis: Act Now or Never

**DOI:** 10.31729/jnma.4939

**Published:** 2020-06

**Authors:** Bishal Yadav

**Affiliations:** 1 Kathmandu Medical College and Teaching Hospital, Sinamangal, Kathmandu, Nepal

The human body fights any infections or foreign bodies through our body’s immune response mechanism.^1^ This immune mechanism is a boon to our body to combat diseases. However, this may cause destructive reactions in our bodies. One such example of a destructive reaction is anaphylaxis. It is an acute, potentially fatal, systemic reaction in which our body responds to some allergens which are usually due to IgE-mediated hypersensitivity reactions resulting from crosslinking of allergen-specific IgE molecules on mast cells or basophils.^2^ The common clinical features patient presents with are urticaria, flushing, pruritis with rashes, difficulty in breathing, hypotension, syncope, dizziness, and other systemic manifestations.^3^ The signs and symptoms usually develop within a few minutes of exposure to the offending agent but can be as late as one hour of post-exposure.^4^

Anaphylaxis is quite a familiar topic among medical students and medical personnel but often overlooked in medical practice. In a study done among dental practitioners in Chennai, none of the participants from the group had a thorough knowledge of all the symptoms of anaphylaxis and only 28% of the practitioners were aware of the correct choice of treatment for anaphylaxis.^5^ In a similar study done to assess the knowledge of diagnosis and management of food-induced anaphylaxis among Russian physician, only 33% of the respondents diagnosed anaphylaxis and of those making a correct diagnosis, only 29% administered the correct choice of treatment.^6^ In a crucial case like anaphylaxis having such small figures of knowledgeable people can cost the medical field a grave fortune.

The reaction can be unpredictable. It can occur due to a small bite of an insect to widely used drugs like antibiotics and non-steroidal anti-inflammatory drugs (NSAIDs) or even random foods like peanuts, tree nuts, fish, milk, eggs, etc.^3^ But luckily, the incidence and fatality rate is lower. Up to five percent of the US population has suffered anaphylaxis and fatal anaphylaxis constitutes less than one percent of total mortality risk.^7^ Despite the low incidence, the consequences of the reaction are severe, so that medical practitioners must be well prepared with the expertise and equipment required to handle the reaction in case of a medical emergency.

Sometimes, during an operative procedure, it is difficult to find an agent that is causing the reaction, and the condition could be fatal if the allergen exposure is not removed early. A similar case was reported in a lady during an elective caesarean section where she was presumed to had an anaphylactic attack due to anaesthesia but was later found to be allergic to the latex used in the urinary catheter. Thus, all the reagents administered around the time of reaction must be used in testing the allergen. Moreover, she was known to her dentist to be allergic to latex but this information was not in her medical notes.^8^ Had there been proper documentation, she wouldn’t have to suffer during her operation. Thus, proper documentation of the allergic history is very important but is often ignored both by the physicians and the patients.

Death of the patient can occur within an hour of anaphylaxis making it a big threat to the patient.^9^ The diagnosis should be early and is primarily based on clinical signs and symptoms and, the other diagnostic modalities could be skin test, in vitro IgE assay, plasma histamine, or serum tryptase.^4^ The lifesaver or the primary management in this fatal condition is intramuscular epinephrine at the mid outer thigh in a dose of 0.01 mg/kg (maximum dose, 0.5 mg) every five to 15 minutes. High flow oxygen to the patients with respiratory symptoms, placing the patients in a recumbent position with lower extremities elevated, fluid resuscitation and vasopressors could be the secondary management of anaphylaxis.^2^ Early identification and management is, therefore, the key to survival in case of anaphylaxis. Anaphylaxis is a threat not only to the patient but is a big threat to the doctor as well.

The doctor should be held accountable for the death of the patient if he/she fails to take the history of the patient properly regarding drugs and allergies before prescribing any medications. In such conditions, it

is undoubtedly a case of negligence because a step taken without precaution can be fatal for the patient. Moreover, in countries like Nepal, anaphylaxis is a threat to doctors also because it is difficult for the doctor to persuade the patient party that death was actually due to the patient’s immune response and not due to incompetence by the doctors. There are also cases where a patient’s party wrongly blames doctors’ negligence over the treatment and instead of seeking legal action, they go violent over doctors and ask for financial compensation.^10^

As medical personnel, we should be prepared to take on any condition that the patient comes with. Nothing should be hidden from our sight or mind hence this makes it very important for us to be aware of anaphylactic reactions and its consequences. Every doctor should properly counsel their patients about the risk of fatal anaphylaxis and refer any patient who has had anaphylaxis to an allergist and immunologist for evaluation. And it is equally the responsibility of each patient and patient party to realize the cause of anaphylaxis and seek medical attention. Apart from this, researches in this matter should be conducted by educational institutions and more effort should be given to take this matter more seriously.

## Conflict of Interest:

**None.**
